# Adolescent neuroticism and short-form video overuse: the mediating roles of depression and anxiety

**DOI:** 10.3389/fpsyg.2026.1777302

**Published:** 2026-09-30

**Authors:** Chang Liu, Jie Wu

**Affiliations:** 1 Student Mental Health Education Center, Jinling Institute of Technology, Nanjing, China; 2 Runxin Studio, Online Ideological and Political Work Center, Jinling Institute of Technology, Nanjing, China; 3 School of Education, Jiangsu Open University, Nanjing, China

**Keywords:** adolescent, anxiety, depression, neuroticism, short-form video overuse

## Abstract

**Background:**

This research examined the association between neuroticism and short-form video overuse (SVO) among adolescents, with particular attention to the mediating roles of depression and anxiety.

**Methods:**

A total of 885 adolescents aged 12 to 18 years completed the Brief Big Five Personality Inventory, the Self-Rating Depression Scale, the Self-Rating Anxiety Scale, and the Short-Form Video Overuse Questionnaire. Based on questionnaire screening, 236 adolescents met the SVO screening criterion and were included in the primary analyses. The same mediation models were also examined in the full sample as supplementary analyses. Pearson correlation, regression analysis, and PROCESS Model 6 were used to test the proposed mediation pathways.

**Results:**

Neuroticism was positively associated with depression, anxiety, and SVO. Mediation analyses showed that the association between neuroticism and SVO was significant through anxiety alone and through the sequential pathway of depression to anxiety, whereas the indirect pathway through depression alone was not significant. In contrast, the sequential pathway of anxiety and depression was not supported.

**Conclusion:**

The findings suggest that depression and anxiety may play different roles in the association between neuroticism and adolescent SVO. In particular, anxiety appeared to be the more proximal emotional factor, whereas depression may contribute more indirectly through its association with anxiety. These results provide a more refined understanding of the emotional processes associated with adolescent SVO and may help inform more targeted prevention and intervention efforts.

## Background

With the growing popularity of short-form video platforms, such content has become a staple in adolescents’ daily routines. However, short-form video overuse (SVO) is increasingly drawing widespread attention (Mu et al., 2022). As primary users of internet applications, adolescents are particularly vulnerable to excessive online engagement (Tian et al., 2018; Lu et al., 2022). By 2023, short-form video platforms such as TikTok had reached 1.8 billion global monthly active users, and these platforms are especially popular among adolescents (Bossen and Kottasz, 2020; Xiong et al., 2024). Previous studies have shown that nearly half of adolescents access short-form video applications daily, with average use exceeding 1 h (Tsitsika et al., 2014). Although moderate use may serve entertainment and social communication purposes, overreliance on such platforms may be associated with a range of psychological and behavioral health challenges (Zhu et al., 2024). Researchers have therefore called for greater attention to the harmful effects of excessive smartphone and digital media use among adolescents, particularly its potential links with mental health and well-being (Elhai et al., 2019; Gligor and Mozoş, 2019; Bai et al., 2024).

In this study, SVO refers to a chronic or recurrent pattern of excessive engagement with short-form video platforms, characterized by impaired control and psychological and behavioral dependence (Qin et al., 2019; Li et al., 2021). This construct should be distinguished from ordinary high-frequency media use, as not all frequent use reflects psychological maladjustment. Rather, SVO emphasizes problematic involvement in short-form video use that may interfere with adolescents’ emotional well-being and daily functioning. This concern is particularly relevant because short-form video platforms combine highly accessible content, continuous personalized recommendations, and rapid reward feedback, all of which may make repeated engagement more difficult to regulate (Becker and Montag, 2019; Bossen and Kottasz, 2020; Xiong et al., 2024).

Adolescents with high neuroticism are considered a high-risk group for SVO (Sha and Dong, 2021; Liu et al., 2021). Neuroticism, as described in the Big Five Personality Theory, is generally characterized by emotional instability and a tendency to experience negative emotions such as anxiety, sadness, and irritability. Importantly, given the widespread adoption of short-form video platforms among adolescents, neuroticism is unlikely to determine whether adolescents engage with these platforms; rather, it may increase vulnerability to excessive and poorly regulated patterns of use. Recent evidence has demonstrated that neuroticism is consistently associated with problematic social media use, suggesting that individuals with higher levels of neuroticism may experience greater difficulties in regulating their digital media behaviors (Meynadier et al., 2024). This vulnerability may be partly related to greater emotional sensitivity and difficulties in regulating negative affect, which may increase the tendency to rely on digital media as a coping strategy when experiencing emotional distress (Gugushvili et al., 2024).

During adolescence, these tendencies may become especially pronounced, as young people face multiple social, emotional, and cognitive challenges during a developmental period marked by still-maturing self-regulation capacities (Li et al., 2023). Therefore, neurotic adolescents may be particularly susceptible to maladaptive patterns of short-form video use, as personality-related vulnerabilities may interact with developmental characteristics and increase the likelihood of excessive digital media engagement (Müller et al., 2018). When emotionally vulnerable adolescents engage excessively with short-form videos, they may experience impaired functioning and a range of mental health problems, which may in turn further reinforce SVO and contribute to a vicious cycle (Humphreys et al., 2017; Mohammed et al., 2020). From the perspective of problematic digital media use, neuroticism may therefore be understood as an important personality-based vulnerability factor that increases adolescents’ susceptibility to maladaptive media engagement (Brand et al., 2019).

Meanwhile, depression and anxiety are two common psychological problems that may play important roles in SVO. Depression is characterized by a persistent state of low mood and reduced interest or pleasure, whereas anxiety typically manifests as excessive worry, tension, and heightened sensitivity to potential threats. Research indicates that highly neurotic individuals, especially adolescents, are more likely to experience depressive symptoms (Sailo and Lalremruati, 2020; Gong et al., 2020). In addition, previous research on personality traits, depression, and TikTok usage disorders has suggested that neuroticism is a significant risk factor for problematic TikTok use, with depression serving as a mediating factor (Montag and Markett, 2024). Similarly, neuroticism has long been recognized as a key factor in the development of anxiety and other common mental disorders (Kotov et al., 2010; Ormel et al., 2013). Research on adolescents has also reaffirmed that neuroticism plays a critical role in predicting anxiety (Liu et al., 2023). Notably, anxiety has been found to positively influence SVO, highlighting its possible role as a more immediate psychological link in this process (Ha, 2023).

The complex relationship among neuroticism, depression, and anxiety provides a theoretical foundation for constructing a serial mediation model. Previous research has shown that mood-related disturbances act as primary mediators linking neurotic personality traits to excessive social media use (Davila et al., 2012; Liu et al., 2023). More directly, Zhang et al. (2024) provided evidence that depression and anxiety may jointly contribute to the association between neuroticism and short-video addiction, underscoring the importance of considering these emotional factors together. Feng et al. (2023) further suggested that anxiety and depression may form a “downward spiral” process in emotionally vulnerable adolescents. Within this reciprocal relationship, depression and anxiety may nevertheless play somewhat different functional roles in the pathway to SVO. Depression may represent a relatively persistent negative affective background associated with low mood, withdrawal, and reduced coping resources, which may increase vulnerability to subsequent anxiety. Longitudinal evidence in youth has also shown that depressive symptoms can predict subsequent increases in certain anxiety symptoms, supporting the plausibility of a depression-to-anxiety pathway during adolescence (Long et al., 2022). Anxiety, in turn, may function as a more proximal emotional state characterized by heightened arousal, worry, and an immediate need for relief.

Because short-form video platforms provide rapid, continuous, and easily accessible stimulation, anxious adolescents may be particularly likely to turn to them for short-term emotional relief, thereby increasing the risk of SVO (Dey et al., 2019; Keles et al., 2020). Accordingly, the depression-to-anxiety sequence represents one theoretically plausible pathway through which neuroticism may be associated with SVO. However, given the close and potentially reciprocal relationship between depression and anxiety, the alternative anxiety-to-depression sequence was also examined for comparison.

Building on this background, the present study examines the association between neuroticism and SVO among adolescents, with particular attention to the mediating roles of depression and anxiety. Given the close interrelations among neuroticism, depression, and anxiety, as well as prior evidence suggesting that mood-related disturbances may help explain problematic digital media use (Davila et al., 2012; Liu et al., 2024; Feng et al., 2023), this study proposes and compares two possible sequential mediation pathways. Specifically, we test whether neuroticism is associated with SVO (1) through the pathway from depression to anxiety, and (2) through the pathway from anxiety to depression. The comparison of these two models was intended to examine which sequential pattern was more consistent with the obsered associations, rather than to infer a definitive temporal ordering between depression and anxiety. The present study seeks to clarify whether depression and anxiety play equivalent or distinct roles in linking neuroticism to SVO. In doing so, the study aims to provide a more precise understanding of the emotional mechanisms associated with adolescent SVO and to offer an empirical basis for more targeted prevention and intervention efforts.

## Materials and methods

### Participants

Using convenience sampling, 885 adolescents between 12 and 18 years old were recruited from two universities and two secondary schools in China, including 546 secondary school students (61.7%) and 339 university students (38.3%). All participants first completed the questionnaire survey. Based on the screening results of the Short-Form Video Overuse Questionnaire, 236 participants (26.7%) were identified as exhibiting SVO and constituted the subgroup used in the primary analyses. To examine whether the findings were dependent on subgroup selection, the same serial mediation analyses were additionally conducted in the full sample of 885 participants. Among these 236 adolescents with SVO, 66 (28.0%) were male and 170 (72.0%) were female.

Participants were recruited from secondary schools and first-year university cohorts to cover the predefined adolescent age range of 12 to 18 years, and all participants met this age criterion. Because the study was designed to examine adolescents as a broadly defined developmental group, eligibility was determined on the basis of whether participants fell within this predefined age range, and exact individual age was therefore not recorded. At the same time, because the sample was obtained through convenience sampling and drawn from different educational settings, it should be regarded as a non-probability adolescent sample. Therefore, the findings should be interpreted with caution when considering their generalizability.

The study was conducted in accordance with the Declaration of Helsinki and approved by the Ethics Committee of Jinling Institute of Technology (Approval No. JLKJ2024No.113). During the research process, questionnaire responses were kept anonymous, and all participants and their legal guardians provided informed consent. The questionnaires were completed in quiet classroom settings or via a secure online platform, with participants free to leave the study at any stage.

### Research instruments

#### Brief big five personality inventory

To assess participants’ neuroticism traits, this study utilized the Chinese version of the Brief Big Five Personality Inventory (Wang et al., 2011). The questionnaire comprises 40 items that assess five core personality traits. In the present study, the neuroticism dimension, which includes 8 items, was used for analysis. Participants rated each item on a 6-point Likert scale ranging from 1 (strongly disagree) to 6 (strongly agree) based on their personal experiences. Higher scores indicated higher levels of neuroticism. In the current sample, the neuroticism subscale demonstrated good internal consistency, with a Cronbach’s alpha of 0.821.

#### Self-rating depression scale (SDS)

The research utilized Zung’s Self-Rating Depression Scale (SDS) to evaluate participants’ levels of depression (Zung, 1965). The scale includes 20 items assessed on a 4-point scale, with response options from “none or a little of the time” to “most or all of the time,” designed to capture participants’ depressive states over a recent period. A higher score reflects more severe depressive symptoms. The depression scale showed acceptable internal reliability in this study, indicated by a Cronbach’s alpha of 0.776.

#### Self-rating anxiety scale (SAS)

The research used Zung’s Self-Rating Anxiety Scale (SAS) to assess participants’ levels of anxiety (Zung, 1971). The scale comprises 20 items rated on a 4-point scale, with options ranging from “none or a little of the time” to “most or all of the time,” aiming to evaluate participants’ recent anxiety states. The anxiety scale exhibited good internal consistency in this study, indicated by a Cronbach’s alpha of 0.857.

#### Short-form video overuse questionnaire

To assess participants’ levels of short-form video overuse (SVO), this study used the Short-Form Video Overuse Questionnaire originally developed by Qin et al. (2019). The questionnaire includes 14 items across four core dimensions: loss of control, withdrawal symptoms, escapism, and reduced productivity. Items are rated on a 5-point Likert scale, higher scores indicating higher levels of SVO. Specifically, items 2, 3, 4, 5, 7, 12 and 13 were used for screening, with “yes” responses scored as 1 and “no” responses scored as 0. The screening procedure was based on the original scale development study by Qin et al. (2019). In that study, eight diagnostic items were initially included, one of which was removed during exploratory factor analysis because of a relatively low factor loading, leaving seven items for screening. Participants with a total score of 4 or higher items were identified as meeting the SVO screening criterion and were included in the subsequent analyses. Qin et al. (2019) suggested that endorsement of four or more of the seven items may indicate a relatively severe tendency toward short-form video addiction, while noting that the exact diagnostic threshold reuired further validation. Accordingly, the ≥ 4 cutoff was treated in the present study as a screening criterion rather that a diagnostic cutoff. The same criterion has also been applied in previous empirical research. For example, Li et al. (2021) used the same criterion to identify short-form video addiction symptoms and reported good internal consistency (Cronbach’ α = 0.91) and satisfactory structural validity for the scale.

In the present study, the questionnaire demonstrated good overall psychometric performance. The Cronbach’s alpha coefficients for the four dimensions were as follows: loss of control (α = 0.560), withdrawal symptoms (α = 0.834), escapism (α = 0.867), and reduced productivity (α = 0.767), with an overall Cronbach’s alpha of 0.888. Additionally, the KMO measure of sampling adequacy was 0.886, and Bartlett’s test of sphericity was significant, indicating strong structural validity and suitability for this study. Although the internal consistency of the loss of control dimension was relatively modest, the overall scale reliability was strong, and therefore the questionnaire was considered appropriate for assessing SVO in this study.

#### Data processing and common method bias test

Data analysis was conducted in SPSS 27.0, correlation analysis, and a common method bias test. Mediation effects were assessed using Hayes’ PROCESS macro (Model 6). The significance of the indirect effects was evaluated using 5000 bootstrap samples with 95% confidence intervals. Gender was included as a covariate in the mediation analyses. In addition to the primary analyses conducted in the SVO-screened subgroup, the same two serial mediation models were repeated in the full sample (*N* = 885), using the continuous SVO total score as the outcome, to examine whether the observed mediation pattern was robust to subgroup selection. Neuroticism scores were also compared between participants who met and did not meet the SVO screening criterion using an independent-samples *t*-test.

To evaluate common method bias, Harman’s single-factor test was conducted (Zhou and Long, 2004). The analysis revealed 17 factors with eigenvalues exceeding 1, with the first factor explaining 11.034% of the variance – significantly below the critical 40% threshold. This suggests that common method bias is minimal in this study, with the data showing strong reliability and validity.

## Results

Table 1 presents the means, standard deviations, and Pearson correlation coefficients for the study variables. Neuroticism, depression, and anxiety are significantly and positively intercorrelated, and all three variables are also significantly and positively correlated with SVO. Gender was not significantly correlated with any of the main study variables.

**TABLE 1:** Means (M), standard deviations(SD), and correlations between variable.

Variables	1	2	3	4	5	M	SD
1 Neuroticism	1					28.62	6.56
2 SVO	0.339***	1				48.52	7.95
3 Depression	0.552***	0.320***	1			52.09	9.10
4 Anxiety	0.585***	0.397***	0.721***	1		47.30	11.47
5 Gender	0.060	0.078	0.099	0.127	1	0.28	0.46

*N* = 236; Gender was coded as 0 for female and 1 for male, with the mean representing the proportion of males.

*p < 0.05,

**p < 0.01,

***p < 0.001.

### Mediation effect test

Pathway 1: Mediating Role of Depression and Anxiety in the Relationship Between Neuroticism and SVO

As shown in Table 2, neuroticism significantly and positively predicted depression, anxiety, and SVO. In the final regression model predicting SVO, anxiety significantly and positively predicted SVO, whereas depression did not significantly predict SVO. Gender was not a significant predictor.

**TABLE 2:** Regression analysis of pathway 1.

Predictor Variables	Depression		Anxiety		SVO	
	β	t	β	t	β	t
Neuroticism	0.761	10.054***	0.470	5.229***	0.191	2.076*
Depression	1.299	1.207	0.715	10.993***	0.023	0.302
Anxiety			1.367	1.275	0.195	3.079**
Gender					0.518	0.499
R^2^	0.556		0.757		0.419	
F	52.185		103.978		12.323	

*p < 0.05,

**p < 0.01,

***p < 0.001.

As shown in Table 3, the indirect effects were further examined using the bias-corrected bootstrap method. The results indicated that the total indirect effect of neuroticism on SVO through depression and anxiety was 0.216, and this mediation effect was significant (see Figure 1).

**TABLE 3:** Testing the indirect effects between neuroticism and SVO (pathway 1).

Mediation path	Effect (Boot)	SE	95% CI	Relative effect
Neuroticism → SVO (Direct Effect)	0.191	0.092	[0.010, 0.371]	
Neuroticism → SVO (Total Indirect Effect)	0.216	0.066	[0.093, 0.354]	100%
1 Depression as a mediator	0.018	0.061	[−0.100, 0.141]	8%
2 Anxietyas a mediator	0.092	0.045	[0.021,0.195]	43%
3 Depression and anxiety as chain mediators	0.106	0.038	[0.033, 0.185]	49%

**FIGURE 1:**
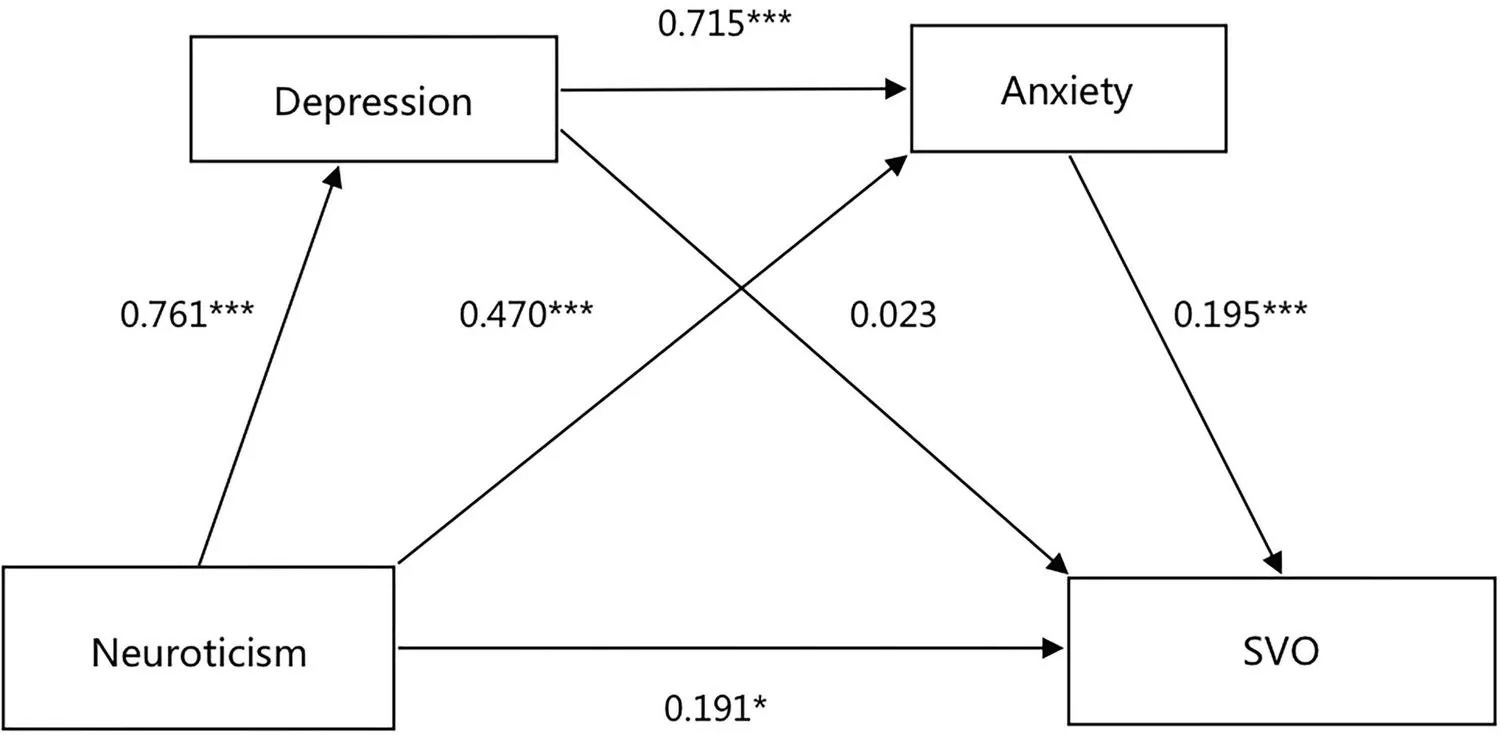
Chain mediation model of depression and anxiety. ***p < 0.001.

Detailed analysis revealed that the mediation effects were generated through the following three pathways:

Neuroticism → Depression → SVO: Effect value = 0.018, 95%CI [−0.100, 0.141], not significant.

Neuroticism → Anxiety → SVO: Effect value = 0.092, 95%CI [0.021, 0.195], significant.

Neuroticism → Depression → Anxiety → SVO: Effect value = 0.106, 95%CI [0.033, 0.185], significant.

Pathway 2: Mediating Role of Anxiety and Depression in the Relationship Between Neuroticism and SVO

As shown in Table 4, neuroticism significantly and positively predicted anxiety, depression, and SVO. In the final regression equation predicting SVO, anxiety remained significantly associated with SVO, whereas depression did not show a significant association with SVO. Gender was not a significant predictor.

**TABLE 4:** Regression analysis of pathway 2.

Predictor variables	Anxiety		Depression		SVO	
	β	t	β	t	β	t
Neuroticism	1.014	10.972***	0.275	3.630***	0.191	2.076*
Anxiety	2.295	1.746	0.479	10.993***	0.195	3.079**
Depression			0.199	0.226	0.023	0.302
Gender					0.518	0.499
R^2^	0.593		0.739		0.419	
F	63.088		92.970		12.323	

*p < 0.05,

**p < 0.01,

***p < 0.001.

As shown in Table 5, the bias-corrected bootstrap analysis indicated that the total indirect effect of neuroticism on SVO through anxiety and depression was 0.216, and the overall mediation effect was significant (see Figure 2).

**TABLE 5:** Testing the indirect effects between neuroticism and SVO (pathway 2).

Mediation path	Effect (Boot)	SE	95%CI	Relative effect
Neuroticism → SVO (Direct Effect)	0.191	0.092	[0.010, 0.371]	
Neuroticism → SVO(Total Indirect Effect)	0.216	0.066	[0.093, 0.354]	100%
1 Anxiety as a mediator	0.198	0.075	[0.058, 0.350]	92%
2 Depression as a mediator	0.006	0.023	[−0.037, 0.056]	3%
3 Anxiety and depression as chain mediators	0.011	0.039	[−0.060, 0.090]	5%

**FIGURE 2:**
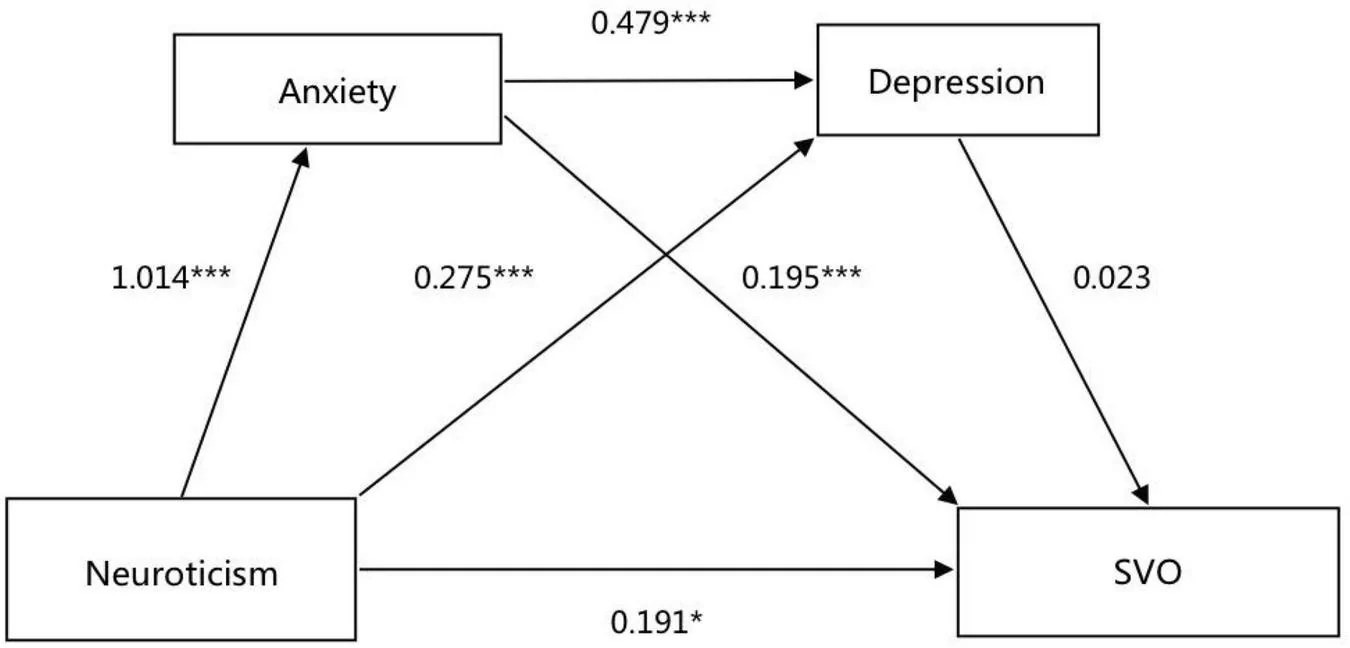
Chain mediation model of anxiety and depression. *p < 0.05, ***p < 0.001.

Detailed analysis revealed that the mediation effects were generated through the following three pathways:

Neuroticism → Anxiety → SVO: Effect value = 0.198,95%CI [0.058, 0.350], significant;

Neuroticism → Depression → SVO: Effect value = 0.006, 95%CI [−0.037, 0.056], not significant;

Neuroticism → Anxiety → Depression → SVO: Effect value = 0.011, 95%CI [−0.060, 0.090], not significant.

These results indicate that, in Pathway 2, only the indirect pathway through anxiety alone was significant, whereas the indirect pathway through depression alone and the sequential pathway of anxiety and depression were not significant.

### Supplementary analyses in the full sample

To examine whether the mediation findings were dependent on the selection of participants meeting the SVO screening criterion, the two serial mediation models were repeated in the full sample (*N* = 885), with gender included as a covariate.

For the depression-to-anxiety model, neuroticism significantly predicted depression (*B* = 0.801, *p* < 0.001), and both neuroticism (*B* = 0.409, *p* < 0.001) and depression (*B* = 0.625, *p* < 0.001) significantly predicted anxiety. In the final model predicting SVO, anxiety was significant (*B* = 0.145, *p* = 0.002), whereas depression was not significant (*B* = 0.067, *p* = 0.173). The direct effect of neuroticism on SVO remained significant (*B* = 0.362, *p* < 0.001). Bootstrap analyses showed significant indirect effects through anxiety alone (*B* = 0.059, 95% CI [0.015, 0.109]) and through the sequential depression-to-anxiety pathway (*B* = 0.072, 95% CI [0.020, 0.125]), whereas the indirect effect through depression alone was not significant (*B* = 0.053, 95% CI [−0.032, 0.141]).

For the alternative anxiety-to-depression model, the indirect effect through anxiety alone was significant (*B* = 0.132, 95% CI [0.036, 0.228]), whereas the indirect effect through depression alone (*B* = 0.018, 95% CI [−0.011, 0.054]) and the sequential anxiety-to-depression pathway (*B* = 0.035, 95% CI [−0.021, 0.094]) were not significant. Thus, the overall pattern observed in the full sample was consistent with that found in the SVO-screened subgroup.

Participants meeting the SVO screening criterion also had significantly higher neuroticism scores (*M* = 28.59, SD = 6.56) than those who did not meet the criterion (*M* = 25.85, SD = 6.94), t(883) = 5.27, *p* < 0.001, 95% CI [1.72, 3.76], Cohen’s d = 0.40.

## Discussion

The present study examined the association between neuroticism and SVO among adolescents, with particular attention to the distinct mediating roles of depression and anxiety. The results showed that neuroticism was positively associated with depression, anxiety, and SVO. More importantly, the association between neuroticism and SVO was supported through anxiety alone and through the sequential pathway of depression to anxiety, whereas the indirect pathway through depression alone was not significant. In contrast, the alternative sequence of anxiety to depression was not supported. Taken together, these findings suggest that negative emotional states should not be treated as a unitary mechanism in adolescent SVO; rather, depression and anxiety may operate at different stages of the emotional pathway linking neuroticism to problematic short-form video use. Importantly, the overall pattern of indirect effects was also observed when the same serial mediation models were repeated in the full sample of 885 participants. Participants meeting the SVO screening criterion also showed significantly higher levels of neuroticism than those who did not meet the criterion. These supplementary findings suggest that the principal mediation pattern was not solely attributable to the selection of the SVO-screened subgroup.

First, the present findings support the view that neuroticism is an important personality-based vulnerability factor for SVO. This is broadly consistent with prior research showing that adolescents high in neuroticism are more prone to emotional instability, depressive symptoms, anxiety, and problematic digital-media use (Gong et al., 2020; Liu et al., 2023; Montag and Markett, 2024). Adolescence is a developmental period marked by heightened emotional reactivity and still-maturing self-regulatory capacities, which may make neurotic adolescents particularly susceptible to maladaptive coping behaviors. In the context of short-form video platforms, this vulnerability may be amplified by platform characteristics such as rapid reward feedback, continuous novelty, personalized recommendation, and extremely easy accessibility (Zhang et al., 2019; Mu et al., 2022). For adolescents who are chronically sensitive to stress and negative affect, such features may make short-form videos especially attractive as a low-effort strategy for distraction and temporary emotional relief.

Second, the findings suggest that depression may function primarily as a more distal emotional factor rather than as an immediate mechanism driving SVO. Although depression was positively associated with both neuroticism and SVO, it did not independently mediate the association between neuroticism and SVO in the final model. This pattern suggests that depressive symptoms may reflect a broader emotional background characterized by negative mood, withdrawal, reduced motivation, and diminished psychological resources, rather than directly translating into repetitive short-form video engagement. In this sense, depression may increase general vulnerability to maladaptive coping while not necessarily triggering SVO in an immediate way. This interpretation is also consistent with recent evidence showing close links between depressive symptoms and problematic short-video or social-media use, while leaving open the possibility that depression exerts its influence through other intervening emotional or behavioral processes (Davila et al., 2012; Zhang et al., 2024; Zhu et al., 2024).

A further possibility is that depressive symptoms may also be related to qualitative characteristics of short-form video engagement, such as emotional content preference. Depression has been associated with cognitive-affective processing biases, including greater attention to and preferential processing of negative or mood-congruent emotional information (Gotlib and Joormann, 2010; Oliver et al., 2019). From this perspective, depressive symptoms may potentially influence preferences for emotionally congruent digital content. However, because the present study did not assess the emotional characteristics of the short-form videos viewed by participants, this interpretation remains tentative and requires further empirical examination.

By contrast, anxiety appeared to be the more proximal emotional mechanism linking neuroticism to SVO. Anxiety is typically characterized by tension, worry, vigilance, and a strong desire for immediate relief from discomfort. Compared with depressive mood, anxiety may be more likely to trigger rapid, avoidance-based behavioral responses, especially in digital environments that provide instant distraction and repeated stimulation (Abend, 2023). Short-form video platforms may be particularly well suited to this process because they offer highly accessible, continuously updated, and algorithmically tailored content that can quickly capture attention and interrupt distressing thoughts (Mu et al., 2022; Xiong et al., 2024). From this perspective, adolescents experiencing anxiety may repeatedly engage with short-form videos not simply because they enjoy the content, but because such platforms provide a readily available means of short-term emotion regulation. This interpretation is in line with previous work suggesting that anxiety is closely related to problematic short-video use and may serve as a key psychological link between stress-related vulnerability and excessive platform engagement (Ha, 2023).

Importantly, this interpretation does not necessarily imply that short-form video use is inherently maladaptive. From the perspective of compensatory internet use theory, individuals may engage with digital media as an attempt to cope with psychological distress, regulate negative emotions, or obtain temporary relief from uncomfortable emotional states (Kardefelt-Winther, 2014). Therefore, short-form video engagement may serve an emotion-regulation function under certain circumstances. However, when adolescents increasingly rely on short-form videos as their primary strategy for managing anxiety and negative affect, this coping pattern may become rigid and difficult to control, thereby increasing the risk of excessive and problematic use (Brand et al., 2019). Thus, the critical issue may not be whether adolescents use short-form videos for emotional regulation, but whether reliance on this strategy becomes increasingly dominant and difficult to regulate.

The significant sequential pathway linking neuroticism to SVO through depression and then anxiety further indicates that depression should not be regarded as irrelevant in the model. Rather, depression may represent an earlier affective condition that increases the likelihood of subsequent anxiety, which then serves as the more immediate predictor of SVO. This interpretation is compatible with research suggesting a dynamic and mutually reinforcing relationship between depressive and anxious states in emotionally vulnerable adolescents (Feng et al., 2023). In other words, neurotic adolescents may experience a more diffuse depressive emotional background, marked by low mood and reduced coping resources, together with or in association with a more activated state of tension, worry, and emotional urgency. It is this more immediate anxious state that may then prompt repetitive short-form video use as an avoidance-based or compensatory coping response. This helps explain why the depression-only pathway was not significant, whereas the sequential pathway through depression and anxiety was supported.

These findings also contribute to the broader literature on problematic digital media use by refining how emotional mechanisms are conceptualized. Previous studies have shown that mood-related disturbances mediate the association between personality traits and excessive social media use (Dey et al., 2019; Miranda et al., 2023; Montag and Markett, 2024). However, the present findings suggest that, at least in the context of adolescent SVO, it may be insufficient to refer to “negative emotion” as a single undifferentiated construct. Instead, depression and anxiety may play functionally distinct roles: depression may index a more distal background vulnerability, whereas anxiety may serve as the more immediate emotional trigger for repetitive and excessive short-form video engagement. This distinction is theoretically important because it aligns more closely with the specific media ecology of short-form video platforms, which emphasize speed, immediacy, novelty, and frictionless consumption. Such features may make SVO especially likely when adolescents are motivated to regulate acute emotional discomfort rather than only persistent low mood.

The present findings also have practical implications. Interventions aimed at reducing adolescent SVO may benefit from focusing not only on screen-time restriction, but also on the emotional functions that short-form video use may serve. In particular, for adolescents high in neuroticism, short-form video use may operate as an easily available strategy for coping with tension, worry, and distress. Therefore, prevention efforts may be more effective when they target anxiety-related emotion regulation difficulties more directly, for example by strengthening adolescents’ skills in stress identification, tolerance of negative affect, and development of alternative offline coping strategies. At the same time, depressive symptoms should not be overlooked, because they may still contribute to SVO indirectly by increasing the likelihood of subsequent anxiety. From a school-based perspective, this means that mental-health programs addressing problematic digital-media use may be more effective when they integrate emotional screening and support, rather than relying solely on behavioral prohibition or digital-discipline education (Bai et al., 2024; Ye et al., 2024).

Several limitations should be acknowledged. First, because this study used a cross-sectional questionnaire design, the findings should be interpreted as associations rather than causal relationships. Although the comparison of the two serial mediation models provides information about which statistical sequence is more consistent with the observed data, it cannot establish the temporal precedence or causal direction between depression and anxiety. Longitudinal or multi-wave designs are therefore needed to clarify how depression and anxiety unfold over time and subsequently relate to SVO. Second, although the primary analyses focused on participants meeting the SVO screening criterion, the same serial mediation models were additionally examined in the full sample and yielded a generally consistent pattern of results. Nevertheless, because the sample was obtained through convenience sampling, caution remains warranted when generalizing the findings to broader adolescent populations. Although all participants were recruited within the predefined age range of 12–18 years, individual-level age information was not collected during questionnaire administration. Therefore, age-related differences in the major study variables could not be examined directly. Future studies should record participants’ exact age and explicitly consider developmental differences across adolescence. Third, all variables were measured through self-report instruments, which may have introduced reporting bias, although the statistical test suggested that common method bias was not serious. Moreover, although the overall psychometric performance of the SVO questionnaire was acceptable, the internal consistency of the loss-of-control dimension was relatively modest, and this should be considered when interpreting the results. Future research could employ longitudinal or multi-wave designs, recruit more diverse adolescent samples, and combine self-report with behavioral or digital-trace indicators to further clarify how personality vulnerability, emotional difficulties, and platform-specific use patterns jointly contribute to SVO.

In conclusion, the present study suggests that the association between neuroticism and adolescent SVO may be better understood through differentiated emotional pathways. Although both depression and anxiety were associated with neuroticism, anxiety emerged as the more immediate emotional factor associated with SVO, whereas depression appeared to play a more indirect role through anxiety. These findings provide a more precise understanding of the emotional processes associated with adolescent SVO and offer an empirical basis for more targeted prevention and intervention efforts.

## Data Availability

Anonymized data are available from the corresponding author upon reasonable request, subject to ethical and confidentiality requirements.
